# Associations between self-reported diabetes and 78 circulating markers of inflammation, immunity, and metabolism among adults in the United States

**DOI:** 10.1371/journal.pone.0182359

**Published:** 2017-07-28

**Authors:** Alison L. Van Dyke, Krystle A. Lang Kuhs, Meredith S. Shiels, Jill Koshiol, Britton Trabert, Erikka Loftfield, Mark P. Purdue, Nicolas Wentzensen, Ruth M. Pfeiffer, Hormuzd A. Katki, Allan Hildesheim, Troy J. Kemp, Ligia A. Pinto, Anil K. Chaturvedi, Mahboobeh Safaeian

**Affiliations:** 1 Division of Cancer Epidemiology and Genetics, National Cancer Institute, National Institutes of Health, Rockville, Maryland, United States of America; 2 Human Papilloma Virus Immunology Laboratory, Leidos Biomedical Research, Inc., Frederick National Laboratory for Cancer Research, Frederick, Maryland, United States of America; University of Nebraska Medical Center, UNITED STATES

## Abstract

Inflammation is increasingly thought to be associated with diabetes; however, only a few inflammation markers have been assessed concurrently in relation to history of diabetes. In the most comprehensive evaluation of inflammation markers and diabetes to date using a Luminex bead-based assay, we measured 78 inflammation-, immune-, and metabolic-related markers detectable in at least 10% of serum samples collected from participants from the Prostate, Lung, Colorectal and Ovarian Cancer (PLCO) screening trial (n = 1,814). At baseline, 6.6% (n = 120) of PLCO participants self-reported a history of diabetes. Cross-sectional associations between these markers and self-reported diabetes were assessed using weighted logistic regression adjusting for sex, smoking status, blood draw age and year, body mass index, and cohort sub-study. Including chemokines [C-C motif ligand (CCL) 19, CCL20, CCL21, C-X-C motif ligand (CXCL) 6, CXCL10, and CXCL11] and soluble cytokine and chemokine receptors [soluble (s) interleukin (IL) 6 receptor (R), soluble tumor necrosis factor receptor (sTNFR) 1, sTNFR2, and sIL-R2], ten inflammation-related markers, were nominally associated with diabetes (*P*<0.05). In addition to these associations, higher levels of insulin, gastric inhibitory polypeptide, and pancreatic polypeptide remained significantly associated with self-reported diabetes with a false discovery rate <5%, indicating that the assay was able to detect markers associated with diabetes. In summary, self-reported diabetes was nominally associated with circulating cytokines, chemokines, and soluble cytokine and chemokine receptors in the most expansive examination of diabetes and inflammation- and immune-related markers to date. These results highlight the need to explore in future prospective studies the role of inflammation markers in diabetes.

## Introduction

Diabetes is associated with significant global health and economic burden. In 2015, 415 million adults (8.3 percent of adults) had diabetes [1]. By 2040, it is projected that 642 million people will live with diabetes. In 2015, five million adults under the age of 80 died as a result of adult-onset diabetes worldwide, and diabetes-related global health expenditures totaled 673 billion in US dollars.

Diabetes is divided into types 1 and 2, which are associated with autoimmune destruction of pancreatic islet beta cells and with insulin resistance, respectively. The more common type 2 diabetes comprises approximately 90 to 95% of adult-onset cases and is linked with numerous health-related and demographic characteristics including obesity, family history of diabetes, older age, physical inactivity, and race and ethnicity [2]. In addition, diabetes is a potential risk factor for numerous complications as well as cancer at multiple sites such as pancreas, liver, biliary tract, breast, colon, rectum, urinary tract, and gynecological tract [1, 3, 4].

Chronic inflammation contributes to cancer risk [5, 6] and may play a role in cancer initiation and promotion among patients with type 2 diabetes and/or obesity [7]. Furthermore, increased levels of inflammation as measured by circulating cytokines and chemokines, white blood cell counts, and inflammatory gene polymorphisms are associated with diabetes [8–10]. These findings suggest that diabetes might contribute to the development of cancer via inflammation. Prior studies of inflammation-related markers and diabetes have measured a limited number of inflammatory markers, primarily C-reactive protein (CRP) and interleukin 6 (IL-6) and, more recently, anti-inflammatory cytokines [10, 11].

The objective of this study was to more broadly evaluate the associations between serologic markers of inflammation and innate immunity and self-reported diabetes using a commercially available panel of 78 inflammation-, immune-, and metabolic-related proteins.

## Materials and methods

### Study population

The Prostate, Lung, Colorectal and Ovarian Cancer Screening Trial (PLCO) is a randomized screening trial that recruited approximately 155,000 participants aged 55–74 years at baseline between 1992 and 2001 [12]. The PLCO study was approved by the Institutional Review Boards at each screening center and at the National Cancer Institute (reference number 358421, protocol number OH97CN041-KKK), and all participants gave written informed consent. Briefly, the objective of the trial was to assess the effect of prostate, lung, colorectal and ovarian cancer screening on disease-specific mortality. In addition to demographic, behavioral, and dietary information, blood samples were obtained at PLCO study baseline and at five subsequent annual visits from participants in the screening arm. Cancer diagnoses were ascertained through annual questionnaires and confirmed by medical chart abstraction and death certificate review. For prostate, lung, colorectal and ovarian cancer, diagnoses were additionally ascertained as a result of clinical follow-up after a positive screening test.

We combined data from three previous PLCO nested case-control studies (i.e., studies of lung cancer [526 cases, 592 matched controls], ovarian cancer [150 cases, 149 matched controls] and non-Hodgkin lymphoma [NHL] [301 cases, 301 matched controls]) that measured serum inflammation-, immune-, and metabolic-related marker levels using a common set of commercially available multiplex panels [6, 13]. Detailed information on the inclusion criteria, matching factors, and inflammation-related markers measured in the lung cancer, ovarian cancer and NHL case-control studies is presented in S1 Table. The combined dataset was limited to non-Hispanic whites (n = 152 excluded). In addition to exclusions made in the original case-control studies, individuals with a personal history of cancer prior to randomization (n = 31) and with incomplete smoking data (n = 11) were also excluded from this study on diabetes and inflammation/immune-related markers, resulting in a total of 1,819 individuals who were included in this analysis. Cancer cases were included in this analysis, as they were cancer-free at the time of blood draw, and represented a small fraction of the data after sampling weights were applied (2.8%).

### Laboratory assay and analysis

Serum specimens used for this study were collected either at baseline (89% of lung, 91% of NHL, and 8% of ovarian studies) or follow up and processed at 1200 xg for 15 minutes, frozen within 2 hours of collection, and stored at -70°C. Time and season of blood draw, but not fasting status, were recorded. These specimens were used to measure circulating levels of 86 markers (77 markers in the lung cancer study, 60 in the ovarian cancer study and 83 in the NHL study; S2 Table) including 4 panels of cytokines and chemokines as well as metabolic disease, soluble receptor, and cardiovascular disease panels. These markers were selected based on a methodologic study that evaluated the performance and reproducibility of multiplexed assays for measurement of inflammation markers in serum [14]. Markers were measured using Luminex bead-based assays (EMD Millipore, Inc., Billerica, MA) according to the manufacturer’s protocols: http://dx.doi.org/10.17504/protocols.io.hbcv62w (cardiovascular panel), http://dx.doi.org/10.17504/protocols.io.hvdb626 (cytokine panel 1a), http://dx.doi.org/10.17504/protocols.io.hveb63e (cytokine panel 1b), http://dx.doi.org/10.17504/protocols.io.hvfb63n (cytokine panel 2), http://dx.doi.org/10.17504/protocols.io.hvgb63w (cytokine panel 3), http://dx.doi.org/10.17504/protocols.io.hvhb636 (metabolic hormone panel), and http://dx.doi.org/10.17504/protocols.io.hvib64e (soluble receptor panel). Concentrations were calculated using either a four- or five-parameter standard curve. Serum samples were assayed in duplicate, and averaged to calculate final concentrations. Blinded duplicates in the lung and NHL studies and duplicate measurements on study subjects in the ovarian cancer study were used to evaluate assay reproducibility through coefficients of variation (CVs) and intraclass correlation coefficients (ICCs) calculated on log-transformed values of the markers. ICCs were >0.8 in 91% of these markers in the lung and NHL studies [6, 13], and in 78% of these markers in the ovarian cancer study [15]. Eight markers with >90% of values below the lowest limit of detection (LLOD) were excluded from all analyses, resulting in 78 evaluable markers. The remaining markers had within-batch CVs <30%.

### Statistical analysis

As described previously in detail [6] to combine data from the case-control studies, we developed sets of propensity-score-adjusted sampling weights to ensure that our analysis accounted for the particular inclusion/exclusion criteria and sampling plan for each study [16] (S1 Table). The sampling weights allowed us to include all participants with marker data (including cancer cases), and made our analysis as representative as possible of the non-Hispanic white PLCO screening arm. Sampling weights were derived from logistic regression models for the probability that an eligible screening arm participant would be selected into any given case-control study. Separate logistic regression models were conducted based on case/control status, study, and sex. Each logistic regression model included age, smoking status, smoking pack-years, and vital status on December 31, 2009. Study-specific weights were then combined for each of the five combinations of case-control studies with a common subset of panels (all 3 studies, lung and NHL, lung and ovary, NHL and ovary, and lung alone). These sampling weights were used in logistic regression models for each dichotomized marker regressed on smoking status and other confounders including age, sex, time of blood draw (PM vs. AM), and study to provide extra control for study-specific selection factors [17]. Simulations suggest that analyses using both weighting methods and additional regression adjustment for matching factors provide a good way to adjust for non-representative sampling in nested case-control studies [18]. While the three nested studies analyzed data from both non-cases and cancer cases, the blood specimens were predominantly drawn at baseline and predated any cancer diagnosis. A sensitivity analysis was also conducted examining relationships between markers and self-reported diabetes among the participants who did not go on to develop cancer.

Information regarding participant characteristics including self-reported diabetes and other prevalent conditions was collected at baseline. Participants were asked to mark Yes or No to answer the following on the baseline questionnaire: “Has a doctor ever told you that you have any of the following conditions?” followed by a list that included “Diabetes.” Type of diabetes and diabetes treatment were not collected. Baseline characteristics of i) the 1,819 individuals with inflammatory marker data included in the current study, ii) the weighted population, and iii) the participants in the PLCO screening arm who met study eligibility criteria were compared. Of the 1,819 participants, five were missing information on self-reported diabetes at baseline.

### Main analysis

The association between each of the 78 markers and self-reported diabetes was estimated in weighted logistic regression models adjusting for smoking status, age at blood draw, sex, BMI category, year of blood draw, and study of origin using standard survey regression analysis software (SAS v9.3, Cary, NC) [17]. A number of markers had a substantial fraction of values below the LLOD, which precluded analysis of these markers as continuous measures. Therefore, separately by study, inflammation marker levels were divided into quantiles (Q), or as detectable and undetectable if >50% of the values were below the LLOD. To assess the trend in distribution across inflammation-related marker quantiles self-reported diabetes (yes vs. no), the *P*-value for the Wald test of the marker as an ordinal variable was examined. We identified all markers where the *P*-value was less than 5%. To account for multiple comparisons, false discovery rate (FDR) criteria of <5% and <10% were applied.

Several restricted analyses were performed to ensure the main findings were not affected by the population in the analyses. These included analyses 1) among individuals with and without self-reported diabetes, who did not develop cancer, 2) excluding the ovarian cancer study participants to eliminate participants with blood draws after baseline, 3) restricted to participants who had their blood drawn in the morning, and 4) including a time of blood draw (PM vs. AM) x inflammation marker multiplicative interaction term and examining the *P*-value for the Wald test of this interaction term. All models were adjusted for smoking status, age at blood draw, sex, BMI category (15 to <25, 25 to <30, 30+ kg/m^2^), year and time (PM vs. AM) of serum collection, and study of origin (i.e., lung cancer, ovarian cancer, or NHL study). In order to identify associations among participants with subclinical diabetes, multivariable linear regression was used to examine potential relationships between inflammation-related markers and metabolic markers, that were strongly associated with self-reported diabetes on logistic regression analysis with *P* < 0.002 (insulin, PP, and GIP), among participants who did not report a history of diabetes. Spearman correlations were used to estimate unweighted correlations between the markers. All analyses were carried out using survey procedures in SAS 9.3 (Cary, NC).

## Results

### Participant characteristics

Compared to the full PLCO screening arm (N = 58,264), individuals with inflammatory marker data included within the current analysis (N = 1,814) were more likely to be male (55.3% vs. 51.4%); older (21.7% vs. 12.7% for those ≥70 years old) and current smokers (22.8% vs. 10.0%) (S3 Table). Following weighting, however, the characteristics of the resulting weighted population closely resembled those of the PLCO screening arm. Following weighting, approximately 6% of individuals had self-reported diabetes (N = 3,332 weighted, N = 120 unweighted). Compared with individuals without a self-reported history of diabetes, those with self-reported diabetes were more likely to be older (31.1% versus 15.4% aged 70 years or older), overweight (40.9% versus 20.1% BMI ≥ 30), former smokers (57.7% versus 42.8%) and to have had a morning blood draw (70% versus 60.9%) (Table 1). Distribution of blood draw time of day (PM vs. AM) did not differ by study [% PM blood draw: 40.8% (lung cancer study), 36.7% (NHL study), 34.1% (ovarian cancer study); χ^2^ *P*-value = 0.08].

**Table 1:** Participant characteristics for the 1,819 individuals with inflammatory marker data and the weighted population, by self-reported diabetes.

Characteristic	Self-Reported Diabetes			
	No		Yes	
	N (%)	Weighted, N (%)	N (%)	Weighted, N (%)
Total	1,694	54,781	120	3,332
Sex				
Female	777 (45.9)	26,763 (48.9)	34 (28.3)	1,493 (44.8)
Male	917 (54.1)	28,018 (51.1)	86 (71.7)	1,839 (55.2)
Age Group (years)				
≤59	320 (18.9)	15,827 (28.9)	14 (11.7)	501 (15.0)
60–64	511 (30.2)	19,644 (35.9)	31 (25.8)	779 (23.4)
65–69	505 (29.8)	10,856 (19.8)	40 (33.3)	1,016 (30.5)
≥70	358 (21.1)	8,453 (15.4)	35 (29.2)	1,035 (31.1)
BMI Category (kg/m^2^)				
≤25	616 (36.4)	17,246 (31.5)	22 (18.3)	761 (22.9)
25-<30	739 (43.6)	25,657 (46.8)	50 (41.7)	1,207 (36.2)
≥30	319 (18.8)	11,034 (20.1)	47 (39.2)	1,363 (40.9)
Smoking Status				
Never	517 (30.5)	25,974 (47.4)	30 (25.0)	1,172 (35.2)
Former	784 (46.3)	23,457 (42.8)	71 (59.2)	1,922 (57.7)
Current	393 (23.2)	5,350 (9.8)	19 (15.8)	238 (7.1)
Original Case-Control Study				
Lung Cancer Study	922 (54.4)	23,477 (42.9)	74 (61.7)	931 (28.0)
Non-Hodgkin Lymphoma Study	529 (31.2)	22,282 (40.7)	40 (33.3)	1,978 (59.4)
Ovarian Cancer Study	243 (14.3)	9,021 (16.5)	6 (5.0)	422 (12.7)
Case-Control Status^a^				
Case	815 (48.1)	645 (1.2)	59 (49.2)	54 (1.6)
Control	879 (51.9)	54,136 (98.8)	61 (50.8)	3,278 (98.4)

After weighting, self-reported diabetes cases were older and had a higher body mass index than did people without a history of self-reported diabetes. Otherwise, participant characteristics were similar. Columns do not add to 100% due to missing data (individuals missing diabetes information, N = 5)

^a^Cases were individuals without cancer at the time of blood collection, but who developed either lung, NHL, or ovarian cancer over the course of follow-up. Controls were free of cancer of interest of each study at the time of selection.

### Analysis of inflammation-, immune-, and metabolic-related markers associated with self-reported diabetes

Out of the 78 different inflammation-, immune- and metabolic-related markers tested, 16 markers were significantly associated with self-reported diabetes at a nominal *P*-value (<0.05) unadjusted for multiple comparisons (Table 2). Following an FDR correction for multiple comparisons, three markers remained significantly associated at the <5% level with a higher odds of self-reported diabetes at baseline: insulin, glucose-dependent insulinotropic peptide (or gastric inhibitory polypeptide; GIP) and pancreatic polypeptide (PP) (Table 2). In addition, higher levels of the cytokine receptors, sIL-6R and sTNFR1, and the chemokines, CCL19, CCL20, CCL21, and CXCL11, were also associated with a history of self-reported diabetes at the <10% level. Of note, there were no participants with self-reported diabetes with insulin levels in the first quantile; subsequently, the first and second quantiles were combined for all analyses. Our results did not materially change in analyses restricted to controls (patients who did not develop cancer in the PLCO screening trial), to baseline serum specimens, or to specimens collected in the AM, as well as analyses adjusted for blood draw time of day (PM vs. AM) in the model.

**Table 2:** Associations between inflammation-, immune-, and metabolic-related markers and self-reported diabetes.

Marker	Quantile (Q)	Total (N)	Self-Reported Diabetes N (%)	POR (95% CI)^a^	*P-trend*
**Insulin**	Q1	193	0	-	
	Q2	198	4 (2.0)	1.00	
	Q3	216	11 (5.1)	2.3 (0.5–10.1)	
	Q4	211	31 (14.7)	12.2 (3.3–45.5)	*0*.*0003*
**GIP**	Q1	196	3 (1.5)	1.00	
	Q2	230	12 (5.2)	4.0 (0.7–22.2)	
	Q3	206	11 (5.3)	4.2 (0.8–21.7)	
	Q4	186	20 (10.8)	15.9 (3.3–77.2)	*0*.*0007*
**PP**	Q1	194	5 (2.6)	1.00	
	Q2	216	7 (3.2)	1.7 (0.3–9.9)	
	Q3	185	14 (7.6)	9.2 (1.9–43.8)	
	Q4	223	20 (9.0)	5.8 (1.2–28.0)	*0*.*001*
sIL-6R	Q1	489	25 (5.1)	1.00	
	Q2	466	28 (6.0)	1.6 (0.6–4.4	
	Q3	373	24 (6.4)	1.3 (0.4–4.4)	
	Q4	485	43 (8.9)	4.3 (1.7–10.6)	*0*.*003*
CCL21	Q1	364	23 (6.3)	1.00	
	Q2	381	20 (5.3)	1.3 (0.4–4.6)	
	Q3	400	31 (7.8)	3.6 (1.2–10.5)	
	Q4	419	40 (9.5)	4.1 (1.4–11.9)	*0*.*004*
CCL20	Q1	521	22 (4.2)	1.00	
	Q2	311	22 (7.1)	0.7 (0.2–2.4)	
	Q3	376	25 (6.6)	1.5 (0.5–4.6)	
	Q4	356	45 (12.6)	4.7 (1.7–12.9)	*0*.*005*
sTNFR1	Q1	458	26 (5.7)	1.00	
	Q2	389	14 (3.6)	0.1 (0.0–0.6)	
	Q3	457	34 (7.4)	1.7 (0.7–4.3)	
	Q4	509	46 (9.0)	2.3 (0.96–5.3)	*0*.*006*
CXCL11	Q1	402	21 (5.2)	1.00	
	Q2	428	34 (7.9)	3.1 (1.2–8.5)	
	Q3	383	26 (6.8)	2.1 (0.7–6.7)	
	Q4	351	33 (9.4)	5.8 (2.1–16.2)	*0*.*007*
CCL19	Q1	405	15 (3.7)	1.00	
	Q2	368	26 (7.1)	1.6 (0.5–5.3)	
	Q3	354	28 (7.9)	1.3 (0.4–4.2)	
	Q4	437	45 (10.3)	4.7 (1.5–15.1)	*0*.*01*
sTNFR2	Q1	403	20 (5.0)	1.00	
	Q2	418	20 (4.8)	0.5 (0.2–1.6)	
	Q3	454	31 (6.8)	0.7 (0.3–1.8)	
	Q4	538	49 (9.1)	2.7 (1.2–6.5)	*0*.*01*
CXCL10	Q1	505	25 (5.0)	1.00	
	Q2	419	19 (4.5)	1.4 (0.5–4.1)	
	Q3	418	29 (6.9)	1.5 (0.6–4.0)	
	Q4	471	47 (10.0)	3.2 (1.3–8.0)	*0*.*02*
CXCL6	Q1	383	19 (5.0)	1.00	
	Q2	378	29 (7.7)	1.1 (0.4–3.1)	
	Q3	402	31 (7.7)	1.0 (0.4–2.8)	
	Q4	401	35 (8.7)	3.3 (1.2–9.1)	*0*.*03*
Amylin	Q1	371	19 (5.1)	1.00	
	Q2	152	6 (3.9)	0.8 (0.2–3.1)	
	Q3	147	11 (7.5)	2.8 (0.9–8.8)	
	Q4	148	10 (6.8)	2.9 (0.9–9.6)	*0*.*04*
sIL-RII	Q1	464	24 (5.2)	1.00	
	Q2	482	24 (5.0)	1.5 (0.6–4.0)	
	Q3	388	26 (6.7)	1.9 (0.7–5.1)	
	Q4	479	46 (9.6)	2.8 (1.1–7.4)	*0*.*04*
Glucagon	Q1	648	31 (4.8)	1.00	
	Q2	170	15 (8.8)	2.7 (1.0–7.0)	
	Q3	-	-	-	
	Q4	-	-	-	*0*.*04*
C-peptide	Q1	197	9 (4.6)	1.00	
	Q2	199	8 (4.0)	0.9 (0.2–3.9)	
	Q3	209	9 (4.3)	0.09 (0.2–3.7)	
	Q4	213	20 (9.4)	3.5 (0.95–13.1)	*0*.*05*
IL-8	Q1	402	23 (5.7)	1.00	
	Q2	425	27 (6.4)	2.1 (0.7–6.0)	
	Q3	487	33 (6.8)	2.2 (0.8–5.9)	
	Q4	504	37 (7.4)	2.6 (1.0–6.3)	*0*.*06*
Leptin	Q1	238	8 (3.4)	1.00	
	Q2	193	11 (5.7)	2.5 (0.5–12.1)	
	Q3	215	17 (7.9)	6.5 (1.2–36.6)	
	Q4	175	10 (5.8)	5.3 (0.7–41.6)	*0*.*07*
EGF	Q1	517	50 (9.7)	1.00	
	Q2	381	27 (7.1)	1.3 (0.5–3.1)	
	Q3	495	29 (5.9)	0.8 (0.3–2.0)	
	Q4	425	14 (3.3)	0.4 (0.1–1.2)	*0*.*08*
CCL13	Q1	368	19 (5.2)	1.00	
	Q2	404	18 (4.5)	1.4 (0.5–4.5)	
	Q3	388	36 (9.3)	1.7 (0.6–5.3)	
	Q4	409	41 (10.1)	2.7 (0.9–7.9)	*0*.*08*
IL-16	Q1	624	47 (7.5)	1.00	
	Q2	295	15 (5.1)	0.6 (0.2–1.7)	
	Q3	307	20 (6.5)	0.9 (0.3–2.5)	
	Q4	343	32 (9.4)	2.0 (0.9–4.5)	*0*.*08*
CCL27	Q1	379	36 (9.6)	1.00	
	Q2	400	27 (6.8)	0.8 (0.3–2.2)	
	Q3	370	32 (8.7)	0.7 (0.3–1.8)	
	Q4	420	19 (4.5)	0.4 (0.1–1.1)	*0*.*09*
CCL8	Q1	443	31 (7.0)	1.00	
	Q2	371	30 (8.1)	2.3 (0.8–6.2)	
	Q3	354	19 (5.4)	2.0 (0.7–5.8)	
	Q4	401	34 (8.5)	2.7 (1.0–7.6)	*0*.*11*
			*P-trend*		
CCL24	Q1	356	18 (5.1)	1.00	
	Q2	445	35 (7.9)	2.1 (0.7–6.3)	
	Q3	357	26 (7.3)	2.0 (0.7–6.1)	
	Q4	411	35 (8.5)	2.5 (0.9–7.1)	*0*.*11*
			*P-trend*	*0*.*11*	
GLP-1	Q1	619	30 (4.9)	1.00	
	Q2	202	16 (7.9)	2.1 (0.8–5.4)	*0*.*13*
	Q3	-	-	-	
	Q4	-	-	-	
			*P-trend*		
sVEGFR2	Q1	358	17 (4.8)	1.00	
	Q2	489	35 (7.2)	1.0 (0.4–2.6)	
	Q3	518	33 (6.4)	1.5 (0.6–3.9)	
	Q4	453	35 (7.8)	2.0 (0.7–5.3)	*0*.*14*
G-CSF	Q1	996	59 (5.9)	1.00	
	Q2	433	31 (7.2)	1.0 (0.4–2.5)	
	Q3	389	30 (7.8)	2.0 (0.9–4.5)	*0*.*14*
	Q4	-	-	-	
IFN-γ	Q1	1019	65 (6.4)	1.00	
	Q2	351	23 (6.6)	1.3 (0.5–3.0)	
	Q3	445	32 (7.2)	1.8 (0.8–3.8)	*0*.*15*
	Q4	-	-	-	
TNF-α	Q1	443	20 (4.7)	1.00	
	Q2	402	33 (8.2)	1.5 (0.5–4.2)	
	Q3	442	24 (5.4)	1.1 (0.4–3.1)	
	Q4	541	43 (8.0)	2.2 (0.8–5.9)	*0*.*17*
CXCL5	Q1	361	18 (5.0)	1.00	
	Q2	417	35 (8.4)	2.6 (0.9–7.2)	
	Q3	333	28 (8.5)	2.1 (0.8–5.9)	
	Q4	458	33 (7.2)	2.7 (0.9–7.7)	*0*.*17*
PYY	Q1	597	28 (4.7)	1.00	
	Q2	118	9 (7.7)	2.6 (0.8–8.8)	
	Q3	106	9 (8.5)	1.9 (0.5–6.9)	*0*.*18*
	Q4	-	-	-	
CXCL9	Q1	386	28 (7.3)	1.00	
	Q2	318	19 (6.0)	0.8 (0.2–2.4)	
	Q3	363	25 (6.9)	1.3 (0.5–3.5)	
	Q4	502	42 (8.4)	1.8 (0.7–4.8)	*0*.*18*
CX3CL1	Q1	1520	97 (6.4)	1.00	
	Q2	298	23 (7.7)	1.7 (0.8–3.8)	*0*.*20*
	Q3	-	-	-	
	Q4	-	-	-	
CXCL13	Q1	332	18 (5.4)	1.00	
	Q2	356	27 (7.6)	1.3 (0.4–4.1)	
	Q3	375	31 (8.3)	1.3 (0.4–4.4)	
	Q4	506	38 (7.5)	2.2 (0.7–7.2)	*0*.*21*
IL-1α	Q1	1489	93 (6.3)	1.00	
	Q2	329	27 (8.2)	1.6 (0.8–3.4)	*0*.*22*
	Q3	-	-	-	
	Q4	-	-	-	
IL-5	Q1	1572	102 (6.5)	1.00	
	Q2	246	18 (7.3)	1.6 (0.7–3.6)	*0*.*24*
	Q3	-	-	-	
	Q4	-	-	-	
IL-17	Q1	890	61 (6.9)	1.00	
	Q2	290	21 (7.2)	0.9 (0.3–2.5)	
	Q3	292	14 (4.8)	0.7 (0.3–1.8)	
	Q4	346	24 (6.9)	1.8 (0.8–4.2)	*0*.*31*
IL-11	Q1	1179	84 (7.2)	1.00	
	Q2	390	30 (7.7)	1.5 (0.7–3.0)	*0*.*32*
	Q3	-	-	-	
	Q4	-	-	-	
CCL15	Q1	340	20 (5.9)	1.00	
	Q2	386	23 (6.0)	0.6 (0.2–1.9)	
	Q3	380	27 (7.1)	1.1 (0.4–2.9)	
	Q4	463	44 (9.5)	1.4 (0.5–4.0)	*0*.*32*
IL-12p40	Q1	1477	95 (6.5)	1.00	
	Q2	341	25 (7.4)	1.4 (0.7–2.9)	*0*.*36*
	Q3	-	-	-	
	Q4	-	-	-	
CCL7	Q1	1551	102 (6.6)	1.00	
	Q2	267	18 (6.8)	0.7 (0.3–1.6)	*0*.*36*
	Q3	-	-	-	
	Q4	-	-	-	
CXCL1,2,3	Q1	479	35 (7.3)	1.00	
	Q2	354	20 (5.7)	1.0 (0.4–2.7)	
	Q3	482	32 (6.6)	1.1 (0.4–2.9)	
	Q4	503	33 (6.6)	1.5 (0.6–3.9)	*0*.*38*
SAA	Q1	235	13 (5.6)	1.00	
	Q2	238	19 (8.0)	0.3 (0.1–1.1)	
	Q3	230	21 (9.1)	1.1 (0.4–3.5)	
	Q4	291	21 (7.2)	0.4 (0.1–1.3)	*0*.*42*
SCF	Q1	841	61 (7.3)	1.00	
	Q2	335	22 (6.6)	0.6 (0.3–1.5)	
	Q3	393	31 (7.9)	0.7 (0.3–1.8)	*0*.*43*
	Q4	-	-	-	
IL-12p70	Q1	1534	96 (6.3)	1.00	
	Q2	287	24 (8.5)	1.4 (0.6–3.2)	*0*.*44*
	Q3	-	-	-	
	Q4	-	-	-	
CCL2	Q1	423	24 (5.7)	1.00	
	Q2	408	32 (7.8)	1.0 (0.4–2.4)	
	Q3	451	28 (6.2)	0.7 (0.3–2.1)	
	Q4	536	36 (6.8)	1.5 (0.6–3.7)	*0*.*44*
IL-1β	Q1	1350	86 (6.4)	1.00	
	Q2	260	19 (7.3)	1.2 (0.5–2.9)	
	Q3	208	15 (7.2)	1.4 (0.6–3.5)	*0*.*44*
	Q4	-	-	-	
sEGFR	Q1	458	27 (5.9)	1.00	
	Q2	456	32 (7.1)	1.1 (0.4–2.9)	
	Q3	517	37 (7.2)	1.6 (0.6–4.3)	
	Q4	387	24 (6.2)	1.3 (0.5–3.3)	*0*.*46*
			*P-trend*		
FGF-2	Q1	1257	79 (6.3)	1.00	
	Q2	284	19 (6.7)	1.1 (0.4–2.8)	
	Q3	277	22 (7.9)	1.4 (0.6–3.2)	*0*.*47*
	Q4	-	-	-	
CCL11	Q1	377	23 (6.1)	1.00	
	Q2	427	25 (5.9)	1.4 (0.6–3.4)	
	Q3	455	32 (7.1)	1.7 (0.6–4.7)	
	Q4	599	40 (7.2)	1.3 (0.5–3.2)	*0*.*47*
TGF-α	Q1	451	33 (7.3)	1.00	
	Q2	390	29 (7.4)	1.5 (0.5–4.2)	
	Q3	470	24 (5.1)	1.1 (0.4–3.1)	
	Q4	507	34 (6.8)	2.2 (0.8–5.9)	*0*.*48*
IL-6	Q1	1287	81 (6.3)	1.00	
	Q2	266	22 (8.3)	1.0 (0.4–2.3)	
	Q3	265	17 (6.4)	1.4 (0.6–3.4)	*0*.*49*
	Q4	-	-	-	
IL-1RA	Q1	1411	92 (6.5)	1.00	
	Q2	407	28 (6.9)	1.3 (0.6–2.6)	*0*.*50*
	Q3	-	-	-	
	Q4	-	-	-	
IL-2	Q1	1392	86 (6.2)	1.00	
	Q2	426	34 (8.0)	1.3 (0.6–2.6)	*0*.*53*
	Q3	-	-	-	
	Q4	-	-	-	
CRP	Q1	214	8 (3.8)	1.00	
	Q2	287	17 (5.9)	1.3 (0.4–4.8)	
	Q3	359	26 (7.2)	1.3 (0.3–4.9)	
	Q4	383	29 (7.6)	0.6 (0.1–3.3)	*0*.*53*
GM-CSF	Q1	1200	77 (6.4)	1.00	
	Q2	303	18 (5.9)	0.7 (0.3–1.8)	
	Q3	315	35 (7.9)	1.5 (0.6–3.4)	*0*.*56*
	Q4	-	-	-	
TSLP	Q1	1243	89 (7.2)	1.00	
	Q2	326	25 (7.7)	0.8 (0.3–1.8)	*0*.*57*
	Q3	-	-	-	
	Q4	-	-	-	
VEGF	Q1	675	40 (5.9)	1.00	
	Q2	339	30 (8.9)	1.5 (0.6–4.0)	
	Q3	360	22 (6.1)	1.2 (0.5–3.0)	
	Q4	444	28 (6.4)	0.7 (0.3–1.9)	*0*.*63*
SAP	Q1	190	10 (5.3)	1.00	
	Q2	216	18 (8.3)	2.4 (0.5–12.4)	
	Q3	248	15 (6.1)	2.2 (0.5–10.0)	
	Q4	340	31 (9.1)	0.9 (0.2–3.9)	*0*.*69*
CCL17	Q1	322	40 (12.5)	1.00	
	Q2	351	17 (4.8)	0.3 (0.1–1.0)	
	Q3	423	25 (5.9)	0.5 (0.2–1.3)	
	Q4	473	32 (6.8)	0.8 (0.3–2.3)	*0*.*70*
sGP130	Q1	455	19 (4.2)	1.00	
	Q2	449	29 (6.5)	1.0 (0.4–2.7)	
	Q3	454	35 (7.7)	0.9 (0.3–2.4)	
	Q4	460	37 (8.1)	1.3 (0.5–3.5)	*0*.*71*
CCL3	Q1	1565	106 (6.8)	1.00	
	Q2	253	14 (5.6)	0.9 (0.4–2.1)	
	Q3	-	-	-	
	Q4	-	-	-	*0*.*74*
IL-10	Q1	1337	85 (6.4)	1.00	
	Q2	283	22 (7.8)	1.0 (0.4–2.3)	
	Q3	198	13 (6.6)	1.2 (0.5–3.0)	
	Q4	-	-	-	*0*.*76*
TRAIL	Q1	418	32 (7.7)	1.00	
	Q2	320	31 (9.8)	0.8 (0.3–2.0)	
	Q3	417	20 (4.8)	0.4 (0.2–1.1)	
	Q4	414	31 (7.5)	1.0 (0.4–2.6)	*0*.*78*
IL-4	Q1	1407	93 (6.6)	1.00	
	Q2	411	27 (6.6)	1.1 (0.5–2.5)	*0*.*78*
	Q3	-	-	-	
	Q4	-	-	-	
			*P-trend*		
IL-7	Q1	1383	93 (6.7)	1.00	
	Q2	435	27 (6.2)	0.9 (0.4–2.1)	*0*.*79*
	Q3	-	-	-	
	Q4	-	-	-	
sVEGFR3	Q1	447	23 (5.2)	1.00	
	Q2	468	31 (6.6)	1.0 (0.4–2.8)	
	Q3	441	35 (8.0)	1.7 (0.7–4.3)	
	Q4	462	31 (6.7)	0.9 (0.3–2.8)	*0*.*83*
CCL22	Q1	416	36 (8.7)	1.00	
	Q2	402	28 (7.0)	1.0 (0.4–2.5)	
	Q3	434	21 (4.9)	0.9 (0.4–2.3)	
	Q4	566	35 (6.2)	0.9 (0.3–2.5)	*0*.*85*
IL-15	Q1	1489	98 (6.6)	1.00	
	Q2	329	22 (6.7)	1.1 (0.5–2.3)	*0*.*86*
	Q3	-	-	-	
	Q4	-	-	-	
CXCL12	Q1	381	29 (7.6)	1.00	
	Q2	441	31 (7.1)	0.7 (0.2–2.2)	
	Q3	401	25 (6.3)	0.5 (0.2–1.4)	
	Q4	346	29 (8.4)	1.3 (0.5–3.2)	*0*.*86*
IFN-α2	Q1	1479	103 (6.5)	1.00	
	Q2	239	17 (7.1)	1.1 (0.4–2.6)	*0*.*88*
	Q3	-	-	-	
	Q4	-	-	-	
CCL4	Q1	517	40 (7.8)	1.00	
	Q2	400	25 (6.3)	0.6 (0.2–1.6)	
	Q3	429	24 (5.6)	0.5 (0.2–1.4)	
	Q4	472	31 (6.6)	1.1 (0.5–2.8)	*0*.*90*
TPO	Q1	990	75 (7.6)	1.00	
	Q2	286	11 (3.9)	1.0 (0.3–2.9)	
	Q3	293	28 (9.6)	1.1 (0.4–2.7)	*0*.*91*
	Q4	-	-	-	
IL-29	Q1	1327	95 (7.2)	1.00	
	Q2	242	19 (7.9)	1.0 (0.4–2.5)	*0*.*92*
	Q3	-	-	-	
	Q4	-	-	-	
sIL-4R	Q1	478	35 (7.4)	1.00	
	Q2	419	26 (6.2)	0.6 (0.2–1.5)	
	Q3	430	32 (7.4)	1.9 (0.8–4.8)	
	Q4	491	27 (5.5)	0.6 (0.3–1.6)	*0*.*93*
TNF-β	Q1	1353	88 (6.5)	1.00	
	Q2	239	17 (7.1)	1.1 (0.5–2.7)	
	Q3	226	15 (6.6)	1.0 (0.4–2.5)	*0*.*93*
	Q4	-	-	-	
sCD40L	Q1	254	21 (8.3)	1.00	
	Q2	244	19 (7.8)	1.5 (0.5–5.0)	
	Q3	1320	80 (6.1)	1.1 (0.5–2.8)	*0*.*94*
	Q4	-	-	-	
IL-33	Q1	1226	81 (6.6)	1	
	Q2	343	33 (9.6)	1.0 (0.4–2.2)	*0*.*99*
	Q3	-	-	-	

Markers in bold, including metabolic-related markers, retained statistical significance of associations with self-reported diabetes with a false discovery rate <5% (*P*≤0.002).

^a^Adjusted for smoking, age at blood draw, gender, BMI, year of blood draw and study of origin

Abbreviations: EGF, epidermal growth factor; VEGF, vascular endothelial growth factor; FGF-2, basic fibroblast growth factor; CCL, chemokine C-C motif ligand; CXCL, chemokine C-X-C motif ligand; IL, interleukin; R, receptor; sVEGFR, soluble vascular endothelial growth factor receptor; sTNFR, soluble tumor necrosis factor receptor; PP, pancreatic polypeptide; sIL-R, soluble interleukin receptor; TRAIL, TNF-related apoptosis-inducing ligand; GIP, gastric inhibitory polypeptide; GM-CSF, granulocyte-macrophage colony-stimulating factor; sEGFR, soluble epidermal growth factor receptor; IFN, interferon; G-CSF, granulocyte colony-stimulating factor; TSLP, thymic stromal lymphopoietin; TPO, thrombopoietin; sGP130, soluble gp130; sCD40L, soluble CD40 ligand; SAP, serum amyloid P; TGF, transforming growth factor; PYY, peptide YY; SCF, stem cell factor; SAA, serum amyloid A; GLP, glucagon-like peptide; POR, prevalence odds ratio; CI, confidence interval; Q, quantile.

Insulin was the marker most strongly associated with self-reported diabetes (Table 2). As there were no people with self-reported diabetes in quantile 1 (Q1), quantile 2 (Q2) was the referent group. Compared with quantile 2 (Q2) of insulin, the adjusted prevalence odds ratios (POR) for diabetes were 2.3 (95% CI: 0.5–10.1) and 12.2 (95% CI: 3.3–45.5) (*P* for trend = 0.0003) for quantiles 3 (Q3) and 4 (Q4), respectively. Compared to individuals in Q1 of GIP; PORs for diabetes were 4.0 (95% CI: 0.7–22.2), 4.2 (95% CI: 0.8–21.7) and 12.2 (95% CI: 3.3–77.2) (*P* for trend = 0.0003) for Q2, Q3 and Q4, respectively. Likewise, compared to individuals in Q1 of PP; PORs for diabetes were 1.7 (95% CI: 0.3–9.9), 9.2 (95% CI: 1.9–43.8) and 5.8 (95% CI: 1.2–28.0) (*P* for trend = 0.001) for Q2, Q3 and Q4, respectively. Following mutual adjustment, both insulin and PP, but not GIP, retained statistical significance. Similar trends were observed for GIP and PP when results were compared by individual study (S4 Table). The association between insulin and diabetes was observed in the NHL study, but not the ovarian cancer study.

### Metabolic marker correlations and relationships among those not self-reporting a history of diabetes with inflammation-related markers associated with diabetes

Insulin, GIP, and PP were only measured in the NHL and ovarian cancer studies; Spearman correlation coefficients were calculated for the three markers by study. Insulin and PP were weakly correlated (0.2 for the NHL and ovarian cancer studies); however, GIP was moderately correlated with insulin (0.5 for the NHL and ovarian cancer studies) and weakly correlated with PP (0.3 for the NHL study and 0.4 for the ovarian cancer study) (Tables A-C in S1 File). Higher insulin levels were associated with higher CCL19, sTNFR1, amylin, and C-peptide levels on linear regression among participants who did not report a history of diabetes and with a self-reported history of diabetes on logistic regression (S5 Table). No other associations were observed between inflammation-related markers and insulin, GIP, or PP among people who did not report a history of diabetes.

## Discussion

In this cross-sectional study, nested within PLCO, we evaluated the largest number of systemic inflammation-, immune-, and metabolic-related markers and self-reported diabetes to date. We found that elevated levels of inflammation-related markers were associated with self-reported diabetes. Of the 78 different inflammation- and metabolic-related markers assessed, 16 were associated with diabetes at *P* <0.05, including glucose transport and signaling pathway proteins (insulin, GIP, PP, glucagon, amylin, C-peptide), cytokines and cytokine receptors (sIL-6R, sTNFR1, STNFR2, and sIL-RII), and chemokines (CCL21, CCL20, CXCL11, CCL19, CXCL10, and CXCL6). As expected, we saw strong associations between insulin, GIP, and PP and self-reported diabetes after correction for multiple comparisons.

### Inflammation and immune-related biomarkers and diabetes

We found significant associations with self-reported diabetes for several circulating cytokines, many of which had not been previously investigated. The literature on this subject has focused largely on a few acute phase reactants and cytokines, such as CRP and IL-6. Higher levels of CRP, high sensitivity CRP (hsCRP), and IL-6 have consistently been associated with an increased risk of insulin resistance [19–21], hyperinsulinism [20, 22], impaired glucose tolerance [23–25], type 2 diabetes [10, 23, 26–30], and metabolic syndrome [31, 32]. Furthermore, CRP, hsCRP, and IL-6 levels decrease following medication and/or lifestyle interventions [26, 33–35].

The lack of association between these markers and self-reported diabetes in our cross-sectional study may be due to several factors including: method of cytokine measurement and/or assay variations, method of diabetes assessment, and differences in study populations. While we observed poor performance of IL-6 in our Luminex bead assay as evidenced by a low level of detection, its soluble receptor, sIL-6R, was associated with a history of self-reported diabetes. Another critical factor may have been the method by which diabetes was assessed. For our study, diabetes was collected by self-report and not according to directly applied clinical criteria.

Given that an estimated 28% of people with diabetes are unaware that they have diabetes [2], this method of outcome measurement may underestimate the actual presence of diabetes in this population. Thus, those with undiagnosed diabetes would have been misclassified as not having diabetes with subsequent bias of the result toward the null. The analysis of associations between metabolic marker levels (insulin, GIP, and PP) and inflammation-related markers among people not reporting a history of diabetes revealed associations between insulin and the chemokine CCL19 and the cytokine receptor sTNFR1, suggesting that there is a portion of participants unaware that they have diabetes. Finally, another important consideration is the population examined. Multiple studies have shown that associations of CRP and IL-6 with type 2 diabetes vary by race and ethnicity [36, 37], sex [27, 30], and body mass index [26, 36, 37].

Using data from prospective studies, pooled estimates of the associations of CRP and IL-6 with diabetes showed a 26 to ~30% increased risk of type 2 diabetes in one meta-analysis [10] with similar results reported in a more recent meta-analysis [38], indicating that inflammation is likely one component in a number of factors contributing to type 2 diabetes. Supporting this assertion are functional data linking circulating IL-6 levels with an inverse association to insulin action that was mediated through adiposity as measured by percent body fat [39]. Finally, even though CRP, hsCRP, and IL-6 were not associated with self-reported diabetes in our study, higher levels of receptors for pro-inflammatory cytokines, including IL-6 (sIL-6R) and tumor necrosis factor-alpha (TNF-α) (sTNFR1 and sTNFR2), showed trends for associations with self-reported diabetes, findings which are supported by prior literature for sIL-6R [19, 25] and either sTNFR1 or sTNFR2 [23, 26, 29, 35].

More recently, studies have begun to examine the relationships between diabetes and other chemokines, anti-inflammatory cytokines, and associated receptors [11]. We observed associations between self-reported diabetes and the chemokines previously reported in the literature, including CXCL10 and interleukin-8 (IL-8) [40], as well as identified a number of novel associations such as: CCL19, CCL20, CCL21, CXCL6, and CXCL11. The mechanism underlying these associations involves a complex interplay between adipocytes and the proinflammatory cytokines and chemokines that they produce, as well as the macrophages recruited to pancreatic islets that communicate via acute phase reactants, chemokines and pro-inflammatory cytokines [41–43].

### Metabolic markers and diabetes

The strong associations between self-reported diabetes and insulin, GIP, and PP found in this study are biologically expected given the critical roles that they play in glucose transport and metabolism. Insulin plays a critical anabolic role in glucose homeostasis and energy storage by facilitating glucose uptake by liver and muscle cells as well as fat uptake by adipocytes and by inhibiting gluconeogenesis, lipolysis, and proteolysis. Hyperinsulinemia contributes to insulin resistance and to the development of type 2 diabetes [44]. GIP is also a regulator of insulin secretion [45]. Patients with type 2 diabetes have been found to have elevated basal GIP in comparison with people who do not have diabetes [46]. Finally, in comparison with people without type 2 diabetes and people with prediabetes, levels of PP among people with type 2 diabetes have been found to be elevated following an oral glucose tolerance test [47]. Furthermore, patients with type 2 diabetes who lost weight following a dietary intervention demonstrated decreased PP, increased insulin secretion, and increased glucose sensitivity [48]. More importantly, hyperinsulinemia and insulin resistance have been associated with both malignant and premalignant conditions [49, 50].

### Strengths and limitations

This study has a number of strengths. While most studies examining inflammation markers and diabetes only measured and analyzed associations between a handful of cytokines and chemokines, this study involved the most comprehensive evaluation of systemic inflammation markers and diabetes to date. It was nested within an established, population-based cohort with standardized specimen collection methods. A large sample size increased the power of the study to detect associations. The strict statistical criteria applied for significance decreased the probability of false positive findings; however, it may have increased the probability of false negative findings and a lack of detection of smaller associations. The strong associations between metabolic-related markers, including insulin, and self-reported diabetes confirms that the assay works.

This study also has limitations. The PLCO study did not collect type of diabetes (type 1 vs. type 2) or information on insulin dependence and other treatments. Also, PLCO did not validate reports of diabetes history. However, as approximately 90 to 95% of cases of diabetes are of type 2 and given that PLCO enrolled adults between the ages of 55 and 74, the vast majority of the diabetes cases included in this analysis are likely type 2. Furthermore, several published studies, including the Women’s Health Initiative (WHI), compare diabetes history by self-report with diagnoses based on medical records. WHI researchers found high positive predictive value of self-reported prevalent and incident diabetes and a high negative predictive value when diabetes is not self-reported [51, 52]. A similar comparison of self-reported diabetes and diagnoses by Canadian administrative health data found kappa coefficients ≥0.80, indicating substantial agreement [53].

Another concern is that a method of marker measurement in addition to the Luminex bead-based assays was not utilized. Major advantages to using such multiplex assays are the reduced sample volume required for the assay and the reduced time to perform the assays. The performance characteristics and sensitivity of these Luminex immune-, inflammation-, and metabolic-related serum markers have been tested and previously reported [14]. Additionally, results from this assay have been extensively analyzed in association with multiple cancer types [6, 15, 54, 55], cigarette smoking [56], body mass index and physical activity [57], and coffee consumption [58]. Thus, the assay methodology used in this study has been extensively evaluated and the results shown to be related to factors impacting systemic inflammation.

Markers also were not measured in all three of the substudies of the PLCO cohort (lung cancer, NHL, and ovarian cancer studies). As such, the study design precludes an assessment of inflammation markers in aggregate. Given our use of baseline data on self-reported diabetes and covariates and inflammation-related markers measured on baseline serum specimens collected predominantly at a single time point, the temporality of the association between the markers and diabetes could not be determined based on cross-sectional data. Analysis restricted to the NHL and lung cancer studies, from which all specimens analyzed were collected at baseline, revealed similar associations between self-reported diabetes and insulin and GIP. While fasting status was not collected in PLCO, analyses restricted to blood draws obtained in the morning yielded similar results. Finally, as the participants in this sample of the PLCO cohort is entirely comprised of Non-Hispanic whites, these results may not be generalizable to people of other races and ethnicities.

### Future directions

A number of future studies can be proposed in light of our findings. The multiplex immune assay, capable of simultaneously measuring markers with varying functions, can be applied to large-scale population-based studies comparing people with and without type 2 diabetes based on clinically documented criteria and incorporating treatment information. Such a design would allow for the examination of the relative strength of associations between type 2 diabetes and glucose metabolism and transport markers vs. that between diabetes and inflammation using an aggregate assessment of systemic inflammation via a calculated score. Given the associations reported in the literature between diabetes and diseases such as cancer, inflammation and cancer, and between diabetes and inflammation, formal mediation analysis in a prospective longitudinal study could provide greater insights into the relative sequence of contributing factors to cancer development.

## Conclusions

In conclusion, we evaluated a large number of systemic inflammation-, immune, and metabolic-related markers among people with self-reported diabetes to date and found that in addition to having strong associations with metabolic markers, self-reported diabetes was also nominally associated with elevated levels of numerous cytokines, chemokines, and their receptors. Additional prospective studies are needed to assess the relationships between inflammation and diabetes to ascertain directionality of the associations and their contributions to the development of chronic diseases such as cancer.

## Supporting information

S1 Table Description of three case-control studies nested within PLCO. (DOC)

S2 Table Markers tested by study. (DOC)

S3 Table Participant characteristics for i) the 1,819 individuals with inflammatory marker data, ii) the weighted population and iii) those in the PLCO screening arm that met the study eligibility criteria. (DOC)

S4 Table Associations between inflammatory and metabolic markers and self-reported diabetes by study. (DOC)

S5 Table Relationships between inflammation-, immune-, and metabolic-related markers and panel markers associated with self-reported diabetes at *P*<0.05. (DOC)

S1 File Spearman correlations for all markers associated with diabetes at the *P*<0.05 level, by study for (Table A) the Lung Study, (Table B) the NHL Study, and (Table C) the Ovarian Study. (DOC)
