# Supplementary material for: Associations between self-reported diabetes and 78 circulating markers of inflammation, immunity, and metabolism among adults in the United States
Source: PLoS One. 2017 Jul 28;12(7):e0182359. doi: 10.1371/journal.pone.0182359 (PMC5533447; doi:10.1371/journal.pone.0182359)
Supplement: S1 Table — (DOC) [file pone.0182359.s001.doc]

**S1 Table**. Description of three case-control studies nested within PLCO.

|  | **Lung cancer** | **NHL** | **Ovarian cancer** |
| --- | --- | --- | --- |
| **Cases (N)** | 526 | 301 | 150 |
| **Controls (N)** | 592 | 301 | 149 |
| **Included in the study (N)** | 998 | 572 | 249 |
| **Proportion of samples from the baseline visit** | 100% | 100% | 9.2% |
| **Median time from blood draw to cancer diagnosis** | 2.9 years | 8 years | 7.9 years |
| **Inclusion criteria** | Screening arm | Screening arm | Females in screening arm |
|  | Baseline questionnaire | Baseline questionnaire | Baseline questionnaire |
|  | Follow-up | Follow-up | Follow-up |
|  | Biochemical consent | Biochemical consent | Biochemical consent |
|  | Valid smoking history | No rare cancer | No rare cancer in controls |
|  | Prior history of cancer | Prior history of cancer | No controls with oophorectomies |
|  | Serum specimens available at baseline | Serum specimens available at baseline | 2+ years pre-diagnosis specimen available |
|  |  |  |  |
| **Matching criteria** | Age at randomization (5-year) | Age at randomization (5-year) | Age at randomization (5-year) |
|  | Gender | Race | Race |
|  | Year of randomization | Gender | Study year of blood draw |
|  | Smoking history | Study center | Year of blood draw |
|  | Pack-years smoked | Entry season/year | Season of blood draw |
|  | Years since quitting smoking | Time of blood draw (am/pm) | Time of blood draw (am/pm) |
| **Panels tested**^1^ | Cytokine panel 1a (22-plex) | Cytokine panel 1a (22-plex) | Cytokine panel 1a (22-plex) |
|  | Cytokine panel 1b (15-plex) | Cytokine panel 1b (15-plex) | Cytokine panel 1b (15-plex) |
|  | Cytokine panel 2 (17-plex) | Cytokine panel 2 (17-plex) | --- |
|  | Cytokine panel 3 (7-plex) | Cytokine panel 3 (7-plex) | --- |
|  | Cardiovascular disease (CVD) panel (3-plex) | --- | CRP only |
|  | Soluble receptor panel (13-plex) | Soluble receptor panel (13-plex) | Soluble receptor panel (13-plex) |
|  | --- | Metabolic hormone panel (9-plex) | Metabolic hormone panel (9-plex) |
| **Total number of markers** | 77 | 83 | 60 |

^1^See Supplemental Table S2 for a list of markers included in each panel
