# Supplementary material for: Associations between self-reported diabetes and 78 circulating markers of inflammation, immunity, and metabolism among adults in the United States
Source: PLoS One. 2017 Jul 28;12(7):e0182359. doi: 10.1371/journal.pone.0182359 (PMC5533447; doi:10.1371/journal.pone.0182359)
Supplement: S2 Table — (DOC) [file pone.0182359.s002.doc]

| **S2 Table: Markers Tested by Study** | | | | |
| --- | --- | --- | --- | --- |
| *Marker*  *Abbreviation* | *Marker Name* | *NHL Study* | *Lung Cancer Study* | *Ovarian Cancer Study* |
| Amylin | Amylin | X |  | X |
| CCL2 | C-C motif ligand 2 | X | X | X |
| CCL3 | C-C motif ligand 3 | X | X | X |
| CCL4 | C-C motif ligand 4 | X | X | X |
| CCL7 | C-C motif ligand 7 | X | X | X |
| CCL8 | C-C motif ligand 8 | X | X |  |
| CCL11 | C-C motif ligand 11 | X | X | X |
| CCL13 | C-C motif ligand 13 | X | X |  |
| CCL15 | C-C motif ligand 15 | X | X |  |
| CCL17 | C-C motif ligand 17 | X | X |  |
| CCL19 | C-C motif ligand 19 | X | X |  |
| CCL20 | C-C motif ligand 20 | X | X |  |
| CCL21 | C-C motif ligand 21 | X | X |  |
| CCL22 | C-C motif ligand 22 | X | X | X |
| CCL24 | C-C motif ligand 24 | X | X |  |
| CCL27 | C-C motif ligand 27 | X | X |  |
| C-peptide | C-peptide | X |  | X |
| CRP | C-reactive protein |  | X | X |
| CX3CL1 | C-X3-C motif ligand 1 | X | X | X |
| CXCL1,2,3 | C-X-C motif ligand 1,2,3 | X | X | X |
| CXCL5 | C-X-C motif ligand 5 | X | X |  |
| CXCL6 | C-X-C motif ligand 6 | X | X |  |
| CXCL9 | C-X-C motif ligand 9 | X | X |  |
| CXCL10 | C-X-C motif ligand 10 | X | X | X |
| CXCL11 | C-X-C motif ligand 11 | X | X |  |
| CXCL12 | C-X-C motif ligand 12 | X | X |  |
| CXCL13 | C-X-C motif ligand 13 | X | X |  |
| EGF | Epidermal growth factor | X | X | X |
| FGF-2 | Basic fibroblast growth factor | X | X | X |
| G-CSF | Granulocyte-colony stimulating factor | X | X | X |
| GIP | Gastric inhibitory polypeptide | X |  | X |
| Glucagon | Glucagon | X |  | X |
| GLP-1 | Glucagon-like peptide 1 | X |  | X |
| GM-CSF | Granulocyte-macrophage colony –stimulating factor | X | X | X |
| IFN-α2 | Interferon alpha 2 | X | X | X |
| IFN-γ | Interferon gamma | X | X | X |
| IL-1α | Interleukin 1 alpha | X | X | X |
| IL-1β | Interleukin 1 beta | X | X | X |
| IL-1RA | Interleukin 1 receptor antagonist | X | X | X |
| IL-2 | Interleukin 2 | X | X | X |
| **Supplemental Table S2: Markers Tested by Study (continued)** | | | | |
| *Marker*  *Abbreviation* | *Marker Name* | *NHL Study* | *Lung Cancer Study* | *Ovarian Cancer Study* |
| IL-4 | Interleukin 4 | X | X | X |
| IL-5 | Interleukin 5 | X | X | X |
| IL-6 | Interleukin 6 | X | X | X |
| IL-7 | Interleukin 7 | X | X | X |
| IL-8 | Interleukin 8 | X | X | X |
| IL-10 | Interleukin 10 | X | X | X |
| IL-11 | Interleukin 11 | X | X |  |
| IL-12(p40) | Interleukin 12 p40 | X | X | X |
| IL-12(p70) | Interleukin 12 p70 | X | X | X |
| IL-15 | Interleukin 15 | X | X | X |
| IL-16 | Interleukin 16 | X | X |  |
| IL-17 | Interleukin 17 | X | X | X |
| IL-29 | Interleukin 29 | X | X |  |
| IL-33 | Interleukin 33 | X | X |  |
| Insulin | Insulin | X |  | X |
| Leptin | Leptin | X |  | X |
| PP | Pancreatic polypeptide | X |  | X |
| PYY | Peptide YY | X |  | X |
| sEGFR | Soluble epidermal growth factor receptor | X | X | X |
| SAA | Serum amyloid a |  | X |  |
| SAP | Serum amyloid p |  | X |  |
| sGP130 | Soluble GP130 | X | X | X |
| sIL-4R | Soluble interleukin receptor 4 | X | X | X |
| sIL-6R | Soluble interleukin receptor 6 | X | X | X |
| sIL-RII | Soluble interleukin receptor 2 | X | X | X |
| sCD40L | Soluble CD40 ligand | X | X | X |
| SCF | Stem cell factor | X | X |  |
| sTNFR1 | Soluble tumor necrosis factor receptor 1 | X | X | X |
| sTNFR2 | Soluble tumor necrosis factor receptor 2 | X | X | X |
| sVEGFR2 | Soluble vascular endothelial growth factor receptor 2 | X | X | X |
| sVEGFR3 | Soluble vascular endothelial growth factor receptor 3 | X | X | X |
| TGF-α | Transforming growth factor alpha | X | X | X |
| TPO | Thrombopoeitin | X | X |  |
| TNF-α | Tumor necrosis factor alpha | X | X | X |
| TNF-β | Tumor necrosis factor beta | X | X | X |
| TRAIL | TNF-related apoptosis-inducing ligand | X | X |  |
| TSLP | Thymic stromal lymphopoeitin | X | X |  |
| VEGF | Vascular endothelial growth factor | X | X | X |
