# Supplementary material for: Associations between self-reported diabetes and 78 circulating markers of inflammation, immunity, and metabolism among adults in the United States
Source: PLoS One. 2017 Jul 28;12(7):e0182359. doi: 10.1371/journal.pone.0182359 (PMC5533447; doi:10.1371/journal.pone.0182359)
Supplement: S3 Table — (DOC) [file pone.0182359.s003.doc]

**S3 Table**: Participant characteristics for i) the 1,819 individuals with inflammatory marker data, ii) the weighted population and iii) those in the PLCO screening arm that met the study eligibility criteria.

|  | N, % | Weighted, N (%) | PLCO Screening Arm, N (%) |
| --- | --- | --- | --- |
| Total | 1,819 | 58,264 | 58,264 |
|  |  |  |  |
| Gender |  |  |  |
| Female | 814 (44.8) | 28,331 (48.6) | 28,331 (48.6) |
| Male | 1,005 (55.3) | 29,933 (51.4) | 29,933 (51.4) |
|  |  |  |  |
| Age group (years) |  |  |  |
| ≤59 | 335 (18.4) | 16,329 (28.0) | 20,056 (34.4) |
| 60-64 | 544 (29.9) | 20,500 (35.2) | 17,954 (30.8) |
| 65-69 | 546 (30.0) | 11,945 (20.5) | 12,877 (22.1) |
| ≥70 | 394 (21.7) | 9,490 (16.3) | 7,377 (12.7) |
|  |  |  |  |
| BMI category (kg/m^2^) |  |  |  |
| <25 | 639 (35.1) | 18,009 (30.1) | 18,914 (32.4) |
| 25-30 | 792 (43.5) | 26,939 (46.2) | 24,753 (42.5) |
| ≥30 | 367 (20.2) | 12,473 (21.4) | 14,015 (24.1) |
| Missing | 21 (1.2) | 843 (1.4) | 582 (1.0) |
|  |  |  |  |
| Smoking status |  |  |  |
| Never | 548 (30.1) | 27,219 (46.7) | 27,389 (47.0) |
| Current | 414 (22.8) | 5,664 (9.7) | 5,843 (10.0) |
| Former | 857 (47.1) | 25,381 (43.6) | 25,032 (43.0) |
|  |  |  |  |
| Diabetes |  |  |  |
| No | 1694 (93.1) | 54,781 (94.3) | 54,185 (93.0) |
| Yes | 120 (6.6) | 3,332 (5.7) | 3,903 (6.7) |
| Unanswered | 5 (0.3) | 0 | 176 (0.3) |
| Original case-control study |  |  |  |
| Lung cancer study | 998 (54.9) | 24,410 (41.9) | --- |
| NHL study | 572 (31.5) | 24,410 (41.9) | --- |
| Ovarian cancer study | 249 (13.7) | 9,444 (16.2) | --- |
|  |  |  |  |
| Cancer case-control Status^a^ |  |  |  |
| Cancer case | 877 (48.2) | 1,637 (2.8) | --- |
| Control | 942 (51.8) | 57,563 (98.8) | --- |
|  |  |  |  |
| ^a^Cases were individuals without cancer at the time of blood collection, but who developed either lung, NHL or ovarian cancer over the course of follow-up. Controls were free of the cancer of interest of each study at the time of selection. | | | |
