# Supplementary material for: Associations between self-reported diabetes and 78 circulating markers of inflammation, immunity, and metabolism among adults in the United States
Source: PLoS One. 2017 Jul 28;12(7):e0182359. doi: 10.1371/journal.pone.0182359 (PMC5533447; doi:10.1371/journal.pone.0182359)
Supplement: S4 Table — (DOC) [file pone.0182359.s004.doc]

| **S4 Table: Associations between Inflammatory and Metabolic Markers and Self-Reported Diabetes by Study.** | | | | | | | |
| --- | --- | --- | --- | --- | --- | --- | --- |
|  |  |  |  |  |  |  |  |
|  |  |  | POR^a^ (95% CI) | | | |  |
|  | Inflammatory Factor | Study | Q1 | Q2 | Q3 | Q4 | *P*-heterogeneity |
|  | Insulin | NHL | - | ref | 2.6 (0.4-17.1) | 31.9 (5.3-192.4) |  |
|  |  | Ovary | - | ref | 0.6 (0.0-12.5) | 1.2 (0.1-19.9) |  |
|  |  | Combined | - | ref | 2.3 (0.5-10.1) | 12.2 (3.3-45.5) | ***0.036*** |
|  |  |  |  |  |  |  |  |
|  | GIP | NHL | ref | 2.2 (0.2-19.5) | 3.5 (0.5-27.9) | 17.5 (2.4-124.9) |  |
|  |  | Ovary | ref | 5.1 (0.1-335.3) | 1.7 (0.0-345.1) | 7.0 (0.1-461.2) |  |
|  |  | Combined | ref | 4.0 (0.7-22.2) | 4.2 (0.8-21.7) | 15.9 (3.3-77.2) | *0.206* |
|  |  |  |  |  |  |  |  |
|  | PP | NHL | ref | 1.2 (0.2-7.4) | 8.9 (1.9-41.5) | 4.2 (0.8-23.5) |  |
|  |  | Ovary | ref | UD | 0.4 (0.0-4.4) | UD |  |
|  |  | Combined | ref | 1.7 (0.3-9.9) | 9.2 (1.9-43.8) | 5.8 (1.2-28.0) | *0.405* |
|  |  |  |  |  |  |  |  |
|  | sIL6R | Lung | ref | 1.3 (0.3-4.8) | 4.1 (1.2-14.1) | 1.3 (0.4-4.4) |  |
|  |  | NHL | ref | 1.4 (0.3-6.1) | 0.7 (0.1-6.3) | 4.7 (1.2-18.4) |  |
|  |  | Ovary | ref | UD | 0.2 (0.2-0.2) | UD |  |
|  |  | Combined | ref | 1.6 (0.6-4.4) | 1.3 (0.4-4.4) | 4.3 (1.7-10.6) | *0.341* |
|  |  |  |  |  |  |  |  |
|  | CCL21 | Lung | ref | 0.8 (0.2-3.2) | 4.3 (1.1-16.4) | 2.2 (0.6-7.9) |  |
|  |  | NHL | ref | 1.6 (0.3-9.1) | 3.2 (0.7-15.4) | 5.3 (1.2-23.4) |  |
|  |  | Combined | ref | 1.3 (0.4-4.6) | 3.6 (1.2-10.5) | 4.1 (1.4-11.9) | *0.461* |
|  |  |  |  |  |  |  |  |
|  | CCL20 | Lung | ref | 2.7 (0.7-10.6) | 2.1 (0.6-7.6) | 5.7 (1.7-19.2) |  |
|  |  | NHL | ref | 0.3 (0.0-1.5) | 1.2 (0.3-5.2) | 4.5 (1.3-16.0) |  |
|  |  | Combined | ref | 0.7 (0.2-2.4) | 1.5 (0.5-4.6) | 4.7 (1.7-12.9) | *0.869* |
|  |  |  |  |  |  |  |  |
|  | sTNFR1 | Lung | ref | 0.1 (0.0-0.3) | 1.1 (0.3-43.2) | 1.6 (0.5-5.4) |  |
|  |  | NHL | ref | 0.4 (0.0-4.0) | 5.6 (1.2-26.8) | 6.1 (1.3-29.3) |  |
|  |  | Ovary | ref | UD | UD | 2.1 (0.1-36.8) |  |
|  |  | Combined | ref | 0.1 (0.0-0.6) | 1.7 (0.7-4.3) | 2.3 (0.96-5.3) | *0.283* |
|  |  |  |  |  |  |  |  |
|  |  |  |  |  |  |  |  |
|  | CXCL11 | Lung | ref | 3.0 (0.9-10.2) | 2.2 (0.6-8.2) | 3.3 (0.9-11.9) |  |
|  |  | NHL | ref | 3.7 (0.9-15.7) | 2.2 (0.4-11.1) | 9.1 (1.9-42.7) |  |
|  |  | Combined | ref | 3.1 (1.2-8.5) | 2.1 (0.7-6.7) | 5.8 (2.1-16.2) | *0.327* |
|  |  |  |  |  |  |  |  |
|  | CCL19 | Lung | ref | 3.6 (0.9-14.8) | 1.5 (0.4-5.5) | 5.3 (1.1-25.0) |  |
|  |  | NHL | ref | 1.1 (0.2-6.2) | 1.3 (0.2-6.3) | 4.2 (0.9-20.1) |  |
|  |  | Combined | ref | 1.6 (0.5-5.3) | 1.3 (0.4-4.2) | 4.7 (1.5-15.1) | *0.704* |
|  |  |  |  |  |  |  |  |
|  | sTNFR2 | Lung | ref | 0.2 (0.0-0.8) | 1.0 (0.3-3.4) | 1.8 (0.5-7.5) |  |
|  |  | NHL | ref | 0.7 (0.1-3.6) | 0.6 (0.1-2.9) | 4.7 (1.3-17.4) |  |
|  |  | Ovary | ref | UD | UD | 2.1 (0.1-36.8) |  |
|  |  | Combined | ref | 0.5 (0.2-1.6) | 0.7 (0.3-1.8) | 2.7 (1.2-6.5) | *0.653* |
|  |  |  |  |  |  |  |  |
|  | CXCL10 | Lung | ref | 1.4 (0.3-6.3) | 2.0 (0.4-8.9) | 1.7 (0.5-6.0) |  |
|  |  | NHL | ref | 0.8 (0.1-5.0) | 1.2 (0.3-5.7) | 4.4 (1.2-16.1) |  |
|  |  | Ovary | ref | 0.1 (UD-32.6) | UD | UD |  |
|  |  | Combined | ref | 1.4 (0.5-4.1) | 1.5 (0.6-4.0) | 3.2 (1.3-8.0) | ***0.01*** |
|  |  |  |  |  |  |  |  |
|  | CXCL6 | Lung | ref | 1.3 (0.4-4.3) | 0.5 (0.1-1.8) | 2.6 (0.8-8.6) |  |
|  |  | NHL | ref | 1.0 (0.2-4.6) | 1.4 (0.3-5.4) | 4.0 (1.0-16.5) |  |
|  |  | Combined | ref | 1.1 (0.4-3.1) | 1.0 (0.4-2.8) | 3.3 (1.2-9.1) | *0.512* |
|  |  |  |  |  |  |  |  |
|  | Amylin | NHL | ref | 0.3 (0.0-2.0) | 3.6 (1.1-11.9) | 4.3 (1.0-17.7) |  |
|  |  | Ovary | ref | UD | UD | UD |  |
|  |  | Combined | ref | 0.8 (0.2-3.1) | 2.8 (0.9-8.8) | 2.9 (0.9-9.6) | ***0.01*** |
|  |  |  |  |  |  |  |  |
|  | sIL-RII | Lung | ref | 3.7 (0.9-15.6) | 3.0 (0.8-11.3) | 3.9 (0.9-15.8) |  |
|  |  | NHL | ref | 1.1 (0.2-5.4) | 2.3 (0.6-8.7) | 3.2 (0.8-12.2) |  |
|  |  | Ovary | ref | 0.9 (0.0-122.7) | 0.6 (0.0-28.1) | 1.0 (0.0-25.2) |  |
|  |  | Combined | ref | 1.5 (0.6-4.0) | 1.9 (0.7-5.1) | 2.8 (1.1-7.4) | *0.726* |
|  |  |  |  |  |  |  |  |
|  | Glucagon | NHL | ref | 2.3 (0.7-7.4) | - | - |  |
|  |  | Ovary | ref | 4.0 (0.4-37.2) | - | - |  |
|  |  | Combined | ref | 2.7 (1.0-7.0) | - | - | *0.682* |
|  |  |  |  |  |  |  |  |
|  | C peptide | NHL | ref | 0.8 (0.2-3.8) | 1.3 (0.2-6.8) | 4.1 (1.0-17.2) |  |
|  |  | Ovary | ref | UD | UD | UD |  |
|  |  | Combined | ref | 0.9 (0.2-3.9) | 0.9 (0.2-3.7) | 3.5 (0.95-13.1) | *0.647* |
|  |  |  |  |  |  |  |  |
|  | ^a^Adjusted for smoking, age at blood draw, gender, BMI, year of blood draw and study of origin | | | | | | |
|  | Abbreviations: POR, prevalence odds ratio; CI, confidence interval; Q, quantile; UD, undefined; GIP, glucose-dependent insulinotropic peptide or gastric inhibitory polypeptide; PP, pancreatic polypeptide; sIL-6R, soluble interleukin 6 receptor; CCL21,  chemokine (C-C motif) ligand 21; CCL20, chemokine (C-C motif) ligand 20; sTNFR1, soluble tumor necrosis factor receptor 1; CXCL11, chemokine (C-X-C motif) ligand 11; CCL19, chemokine (C-C motif) ligand 19; sTNFR2, soluble tumor necrosis factor receptor 2; CXCL10,chemokine (C-X-C motif) ligand 10; CXCL6, chemokine (C-X-C) ligand 6; sIL-RII, soluble interleukin 2 receptor | | | | | | |
