# Supplementary material for: Associations between self-reported diabetes and 78 circulating markers of inflammation, immunity, and metabolism among adults in the United States
Source: PLoS One. 2017 Jul 28;12(7):e0182359. doi: 10.1371/journal.pone.0182359 (PMC5533447; doi:10.1371/journal.pone.0182359)
Supplement: S5 Table — (DOC) [file pone.0182359.s005.doc]

| **S5 Table - Relationships between Inflammation-, Immune-, and Metabolic-Related Markers and Panel Markers Associated with Self-Reported Diabetes at *P*<0.05** | | | | | | |
| --- | --- | --- | --- | --- | --- | --- |
| **Marker** | **Quantile (Q)** | **Total (N)** | **Self-Reported Diabetes**  **N (%)** | **Insulin among**  **Those without Self-Reported Diabetes**  **Mean (Standard Error)**^a^ | **GIP among**  **Those without Self-Reported Diabetes**  **Mean (Standard Error)**^a^ | **PP among**  **Those without Self-Reported Diabetes**  **Mean (Standard Error)**^a^ |
| **Insulin** | Q1  Q2  Q3  Q4 | 193  198  216  211 | 0  4 (2.0)  11 (5.1)  31 (14.7)  *P-value* | N/A | 33 (4)  39 (5)  63 (8)  110 (9)  *<0.0001* | 155 (21)  153 (19)  173 (23)  252 (56)  *0.35* |
| **GIP** | Q1  Q2  Q3  Q4 | 196  230  206  186 | 3 (1.5)  12 (5.2)  11 (5.3)  20 (10.8)  *P-value* | 86 (86)  456 (94)  667 (80)  1,695 (267)  *<0.0001* | N/A | 157 (25)  185 (46)  164 (18)  224 (20)  *0.04* |
| **PP** | Q1  Q2  Q3  Q4 | 194  216  185  223 | 5 (2.6)  7 (3.2)  14 (7.6)  20 (9.0)  *P-value* | 503 (123)  783 (110)  700 (130)  1,154 (237)  *0.06* | 33 (5)  60 (8)  59 (7)  83 (9)  *<0.0001* | N/A |
| sIL-6R | Q1  Q2  Q3  Q4 | 489  466  373  485 | 25 (5.1)  28 (6.0)  24 (6.4)  43 (8.9)  *P-value* | 717 (88)  757 (116)  737 (85)  1,037 (214)  *0.61* | 62 (7)  61 (9)  56 (7)  64 (8)  *0.83* | 179 (18)  199 (43)  168 (21)  195 (23)  *0.71* |
| CCL21 | Q1  Q2  Q3  Q4 | 364  381  400  419 | 23 (6.3)  20 (5.3)  31 (7.8)  40 (9.5)  *P-value* | 546 (115)  863 (241)  522 (115)  700 (115)  *0.44* | 34 (5)  41 (7)  34 (6)  47 (7)  *0.45* | 158 (26)  173 (41)  149 (23)  128 (17)  *0.62* |
| CCL20 | Q1  Q2  Q3  Q4 | 521  311  376  356 | 22 (4.2)  22 (7.1)  25 (6.6)  45 (12.6)  *P-value* | 543 (121)  570 (103)  554 (142)  1,072 (361)  *0.68* | 35 (5)  52 (8)  37 (7)  37 (6)  *0.33* | 153 (22)  125 (20)  104 (23)  221 (67)  *0.17* |
| sTNFR1 | Q1  Q2  Q3  Q4 | 458  389  457  509 | 26 (5.7)  14 (3.6)  34 (7.4)  46 (9.0)  *P-value* | 605 (80)  687 (84)  1,043 (196)  917 (107)  *0.01* | 54 (7)  58 (7)  51 (8)  71 (10)  *0.50* | 196 (20)  154 (19)  217 (44)  169 (18)  *0.19* |
| CXCL11 | Q1  Q2  Q3  Q4 | 402  428  383  351 | 21 (5.2)  34 (7.9)  26 (6.8)  33 (9.4)  *P-value* | 513 (92)  690 (139)  892 (247)  602 (85)  *0.27* | 44 (7)  41 (6)  40 (7)  31 (4)  *0.28* | 162 (26)  180 (42)  100 (19)  151 (23)  *0.09* |
| CCL19 | Q1  Q2  Q3  Q4 | 405  368  354  437 | 15 (3.7)  26 (7.1)  28 (7.9)  45 (10.3)  *P-value* | 420 (95)  694 (99)  539 (115)  980 (255)  *0.03* | 37 (6)  50 (7)  33 (6)  37 (5)  *0.37* | 139 (28)  185 (44)  135 (22)  145 (16)  *0.85* |
| sTNFR2 | Q1  Q2  Q3  Q4 | 403  418  454  538 | 20 (5.0)  20 (4.8)  31 (6.8)  49 (9.1)  *P-value* | 696 (100)  759 (93)  804 (128)  1,014 (290)  *0.64* | 69 (9)  53 (6)  61 (8)  57 (7)  *0.34* | 185 (24)  155 (17)  204 (45)  194 (27)  *0.31* |
| CXCL10 | Q1  Q2  Q3  Q4 | 505  419  418  471 | 25 (5.0)  19 (4.5)  29 (6.9)  47 (10.0)  *P-value* | 706 (96)  730 (131)  699 (104)  1,152 (289)  *0.53* | 72 (8)  51 (8)  54 (8)  57 (7)  *0.21* | 218 (33)  168 (20)  159 (23)  171 (22)  *0.51* |
| CXCL6 | Q1  Q2  Q3  Q4 | 383  378  402  401 | 19 (5.0)  29 (7.7)  31 (7.7)  35 (8.7)  *P-value* | 575 (105)  520 (96)  879 (229)  638 (130)  *0.36* | 41 (5)  36 (6)  38 (5)  44 (8)  *0.84* | 139 (21)  185 (45)  132 (21)  163 (25)  *0.63* |
| Amylin | Q1  Q2  Q3  Q4 | 371  152  147  148 | 19 (5.1)  6 (3.9)  11 (7.5)  10 (6.8)  *P-value* | 253 (104)  449 (70)  735 (109)  2,142 (343)  *<0.0001* | 31 (4)  53 (8)  76 (11)  110 (10)  *<0.0001* | 132 (18)  211 (30)  172 (26)  262 (72)  *0.04* |
| sIL-RII | Q1  Q2  Q3  Q4 | 464  482  388  479 | 24 (5.2)  24 (5.0)  26 (6.7)  46 (9.6)  *P-value* | 700 (87)  772 (120)  727 (94)  1,039 (192)  *0.32* | 54 (6)  57 (8)  60 (7)  71 (9)  *0.36* | 195 (22)  174 (21)  217 (43)  154 (18)  *0.36* |
| Glucagon | Q1  Q2  Q3  Q4 | 648  170  -  - | 31 (4.8)  15 (8.8)  -  -  *P-value* | 745 (78)  1,018 (129)  -  -  *0.048* | 52 (5)  93 (11)  -  -  *0.0003* | 152 (14)  292 (66)  -  -  *0.05* |
| C-peptide | Q1  Q2  Q3  Q4 | 197  199  209  213 | 9 (4.6)  8 (4.0)  9 (4.3)  20 (9.4)  *P-value* | 235 (90)  438 (83)  591 (110)  1,874 (255)  *<0.0001* | 29 (4)  43 (6)  56 (7)  109 (9)  *<0.0001* | 132 (18)  186 (26)  156 (23)  249 (52)  *0.07* |

Markers are listed in order of lowest to highest *P*-value. Markers in bold are those that retained statistical significance after a FDR correction (<5%). ^a^Adjusted for smoking status, age at blood draw, sex, BMI category, year of blood draw, and study of origin. Abbreviations: POR, prevalence odds ratio; CI, confidence interval; Q, quantile; GIP, glucose-dependent insulinotropic peptide or gastric inhibitory polypeptide; PP, pancreatic polypeptide; sIL-6R, soluble interleukin 6 receptor; CCL21, chemokine (C-C motif) ligand 21; CCL20, chemokine (C-C motif) ligand 20; sTNFR1, soluble tumor necrosis factor receptor 1; CXCL11, chemokine (C-X-C motif) ligand 11; CCL19, chemokine (C-C motif) ligand 19; sTNFR2, soluble tumor necrosis factor receptor 2; CXCL10,chemokine (C-X-C motif) ligand 10; CXCL6, chemokine (C-X-C) ligand 6; sIL-RII, soluble interleukin 2 receptor.
