# Supplementary material for: Associations between self-reported diabetes and 78 circulating markers of inflammation, immunity, and metabolism among adults in the United States
Source: PLoS One. 2017 Jul 28;12(7):e0182359. doi: 10.1371/journal.pone.0182359 (PMC5533447; doi:10.1371/journal.pone.0182359)
Supplement: S1 File — (DOC) [file pone.0182359.s006.doc]

| **Supplemental S1 File**: Spearman correlations for all markers associated with diabetes at the *P*<0.05 level, by study | | | | | | | | | | | | | | | | | |
| --- | --- | --- | --- | --- | --- | --- | --- | --- | --- | --- | --- | --- | --- | --- | --- | --- | --- |
|  | |  |  |  |  |  |  |  |  |  |  |  |  |  |  |  |  |
| **Table A.** Lung Study | | |  |  |  |  |  |  |  |  |  |  |  |  |  |  |  |
|  | Insulin | | GIP | PP | sIL-6R | CCL21 | CCL20 | sTNFR1 | CXCL11 | CCL19 | sTNFR2 | CXCL10 | CXCL6 | Amylin | sIL-RII | Glucagon | C-peptide |
| Insulin | - | |  |  |  |  |  |  |  |  |  |  |  |  |  |  |  |
| GIP | - | | - |  |  |  |  |  |  |  |  |  |  |  |  |  |  |
| PP | - | | - | - |  |  |  |  |  |  |  |  |  |  |  |  |  |
| sIL-6R | - | | - | - | 1.0 |  |  |  |  |  |  |  |  |  |  |  |  |
| CCL21 | - | | - | - | 0.2 | 1.0 |  |  |  |  |  |  |  |  |  |  |  |
| CCL20 | - | | - | - | 0.1 | 0.2 | 1.0 |  |  |  |  |  |  |  |  |  |  |
| sTNFR1 | - | | - | - | 0.5 | 0.3 | 0.1 | 1.0 |  |  |  |  |  |  |  |  |  |
| CXCL11 | - | | - | - | 0.3 | 0.1 | 0.1 | 0.3 | 1.0 |  |  |  |  |  |  |  |  |
| CCL19 | - | | - | - | 0.3 | 0.3 | 0.2 | 0.3 | 0.3 | 1.0 |  |  |  |  |  |  |  |
| sTNFR2 | - | | - | - | 0.5 | 0.3 | 0.2 | 0.8 | 0.3 | 0.4 | 1.0 |  |  |  |  |  |  |
| CXCL10 | - | | - | - | 0.3 | 0.1 | 0.1 | 0.2 | 0.5 | 0.4 | 0.4 | 1.0 |  |  |  |  |  |
| CXCL6 | - | | - | - | 0.3 | 0.1 | 0.2 | 0.3 | 0.5 | 0.4 | 0.3 | 0.3 | 1.0 |  |  |  |  |
| Amylin | - | | - | - | - | - | - | - | - | - | - | - | - | 1.0 |  |  |  |
| sIL-RII | - | | - | - | 0.5 | 0.2 | 0.1 |  | 0.2 | 0.3 | 0.5 | 0.2 | 0.3 | - | 1.0 |  |  |
| Glucagon | - | | - | - | - | - | - | - | - | - | - | - | - | - | - | 1.0 |  |
| C-peptide | - | | - | - | - | - | - | - | - | - | - | - | - | - | - | - | 1.0 |
|  |  | |  |  |  |  |  |  |  |  |  |  |  |  |  |  |  |

| **Table B.** NHL Study | |  |  |  |  |  |  |  |  |  |  |  |  |  |  |  |
| --- | --- | --- | --- | --- | --- | --- | --- | --- | --- | --- | --- | --- | --- | --- | --- | --- |
|  | Insulin | GIP | PP | sIL-6R | CCL21 | CCL20 | sTNFR1 | CXCL11 | CCL19 | sTNFR2 | CXCL10 | CXCL6 | Amylin | sIL-RII | Glucagon | C-peptide |
| Insulin | 1.0 |  |  |  |  |  |  |  |  |  |  |  |  |  |  |  |
| GIP | 0.5 | 1.0 |  |  |  |  |  |  |  |  |  |  |  |  |  |  |
| PP | 0.2 | 0.3 | 1.0 |  |  |  |  |  |  |  |  |  |  |  |  |  |
| sIL-6R | 0.1 | 0.0 | 0.1 | 1.0 |  |  |  |  |  |  |  |  |  |  |  |  |
| CCL21 | 0.1 | 0.1 | 0.1 | 0.0 | 1.0 |  |  |  |  |  |  |  |  |  |  |  |
| CCL20 | 0.2 | 0.0 | 0.0 | 0.1 | 0.2 | 1.0 |  |  |  |  |  |  |  |  |  |  |
| sTNFR1 | 0.2 | 0.0 | 0.0 | 0.3 | 0.2 | 0.1 | 1.0 |  |  |  |  |  |  |  |  |  |
| CXCL11 | 0.1 | 0.0 | 0.0 | 0.2 | 0.2 | 0.2 | 0.2 | 1.0 |  |  |  |  |  |  |  |  |
| CCL19 | 0.2 | 0.0 | 0.1 | 0.1 | 0.3 | 0.3 | 0.3 | 0.4 | 1.0 |  |  |  |  |  |  |  |
| sTNFR2 | 0.1 | 0.0 | 0.0 | 0.2 | 0.2 | 0.2 | 0.6 | 0.3 | 0.3 | 1.0 |  |  |  |  |  |  |
| CXCL10 | 0.0 | 0.0 | 0.0 | 0.1 | 0.1 | 0.1 | 0.2 | 0.5 | 0.4 | 0.4 | 1.0 |  |  |  |  |  |
| CXCL6 | 0.1 | 0.0 | 0.0 | 0.2 | 0.2 | 0.1 | 0.2 | 0.5 | 0.3 | 0.2 | 0.1 | 1.0 |  |  |  |  |
| Amylin | 0.7 | 0.4 | -0.1 | 0.2 | 0.1 | 0.1 | 0.2 | 0.0 | 0.0 | 0.1 | 0.0 | 0.0 | 1.0 |  |  |  |
| sIL-RII | 0.2 | 0.1 | 0.1 | 0.3 | 0.1 | 0.0 | 0.2 | 0.1 | 0.1 | 0.1 | 0.0 | 0.1 | 0.2 | 1.0 |  |  |
| Glucagon | 0.2 | 0.1 | 0.0 | 0.1 | -0.3 | 0.0 | 0.1 | 0.0 | -0.2 | -0.1 | 0.0 | 0.0 | 0.2 | 0.0 | 1.0 |  |
| C-peptide | 0.8 | 0.5 | 0.2 | 0.1 | 0.1 | 0.2 | 0.2 | 0.1 | 0.1 | 0.1 | 0.1 | 0.1 | 0.8 | 0.2 | 0.2 | 1.0 |
|  |  |  |  |  |  |  |  |  |  |  |  |  |  |  |  |  |

| **Table C.** Ovary Study | |  |  |  |  |  |  |  |  |  |  |  |  |  |  |  |
| --- | --- | --- | --- | --- | --- | --- | --- | --- | --- | --- | --- | --- | --- | --- | --- | --- |
|  | Insulin | GIP | PP | sIL-6R | CCL21 | CCL20 | sTNFR1 | CXCL11 | CCL19 | sTNFR2 | CXCL10 | CXCL6 | Amylin | sILRII | Glucagon | C-peptide |
| Insulin | 1.0 |  |  |  |  |  |  |  |  |  |  |  |  |  |  |  |
| GIP | 0.5 | 1.0 |  |  |  |  |  |  |  |  |  |  |  |  |  |  |
| PP | 0.2 | 0.4 | 1.0 |  |  |  |  |  |  |  |  |  |  |  |  |  |
| sIL-6R | 0.1 | 0.0 | 0.1 | 1.0 |  |  |  |  |  |  |  |  |  |  |  |  |
| CCL21 | - | - | - | - | - |  |  |  |  |  |  |  |  |  |  |  |
| CCL20 | 0.2 | - | - | - | - | - |  |  |  |  |  |  |  |  |  |  |
| sTNFR1 | 0.2 | 0.0 | 0.0 | 0.3 | - | - | 1.0 |  |  |  |  |  |  |  |  |  |
| CXCL11 | - | - | - | - | - | - | - | - |  |  |  |  |  |  |  |  |
| CCL19 | - | - | - | - | - | - | 0.3 | - | - |  |  |  |  |  |  |  |
| sTNFR2 | 0.1 | 0.1 | 0.0 | 0.3 | - | - | 0.6 | - | - | 1.0 |  |  |  |  |  |  |
| CXCL10 | 0.0 | 0.1 | 0.1 | 0.3 | - | - | 0.2 | - | - | 0.6 | 1.0 |  |  |  |  |  |
| CXCL6 | - | - | - | - | - | - | - | - | - | - | - | 1.0 |  |  |  |  |
| Amylin | 0.7 | 0.4 | -0.1 | 0.2 | - | - | 0.2 | - | - | 0.1 | 0.0 | 0.0 | 1.0 |  |  |  |
| sIL-RII | 0.2 | 0.0 | -0.1 | 0.4 | - | - | 0.2 | - | - | 0.3 | 0.1 | - | 0.2 | 1.0 |  |  |
| Glucagon | 0.2 | 0.1 | 0.0 | 0.1 | - | - | 0.1 | - | - | -0.1 | 0.0 | 0.0 | 0.2 | 0.0 | 1.0 |  |
| C-peptide | 0.8 | 0.5 | 0.2 | 0.1 | - | - | 0.2 | - | - | 0.1 | 0.1 | 0.1 | 0.8 | 0.2 | 0.2 | 1.0 |
